# Repurposing mitochondrial-targeting drugs for management of ICANS in CAR T-cell therapy: a novel steroid-sparing approach

**DOI:** 10.3389/fphar.2026.1896516

**Published:** 2026-07-28

**Authors:** Atena Zahedi, Onwodi V. Ifejeokwu, Shawn P. Griffin, Erin A. Dean

**Affiliations:** 1 Department of Clinical Pharmacy Practice, University of California Irvine, Irvine, CA, United States; 2 Department of Anatomy and Neurobiology, University of California Irvine, Irvine, CA, United States; 3 Hematopoietic Stem Cell Transplantation and Cellular Therapy Program, Division of Hematology/Oncology, Department of Medicine, University of California Irvine, Orange, CA, United States

**Keywords:** CAR T-cell therapy, drug repurposing, hematologic malignancies, ICANS, leflunomide, mitochondria, neuroinflammation, steroid-sparing

## Abstract

Chimeric antigen receptor (CAR) T-cell therapy has transformed the treatment landscape for relapsed and refractory hematologic malignancies. However, immune effector cell-associated neurotoxicity syndrome (ICANS) remains one of the most clinically significant and potentially life-threatening toxicities, limiting the broader applicability of this otherwise promising modality. Current standard-of-care (SOC) management of ICANS relies heavily on corticosteroids, which are not always effective and at high doses and with prolonged exposure may carry substantial risks for infectious complications, steroid-induced hyperglycemia, acute steroid myopathy, and steroid-induced psychosis. Steroid-sparing strategies are thus urgently needed. In this article, we emphasize the role of immune cell activation and mitochondrial dysfunction as a feedforward amplifier of neuroinflammation during ICANS and describe a structured framework for identifying and selecting mitochondrial-targeting drugs suitable for repurposing in ICANS prophylaxis and treatment. These agents are recommended based on mechanistic relevance, central nervous system bioavailability, oncological safety, and translational precedent. We apply this framework to generate a candidate landscape and highlight leflunomide as a prototype example. This work details leflunomide’s dihydroorotate dehydrogenase-centered mitochondrial pharmacology, summarizes evidence from adjacent neuroinflammatory disease contexts, and outlines a proposed staged clinical evaluation pathway.

## 1 Introduction

CAR T-cell therapy has achieved remarkable outcomes in patients with certain B-cell malignancies refractory to conventional treatments. Landmark approvals of CD19-directed and BCMA-directed products have validated the platform, yet broad clinical implementation continues to be constrained by treatment-emergent toxicities, most notably cytokine release syndrome (CRS) and immune effector cell-associated neurotoxicity syndrome (ICANS).

ICANS is characterized by a spectrum of neurological manifestations—ranging from mild confusion, word-finding difficulty, and tremor to severe encephalopathy, seizure, cerebral edema, and death (Aziz et al., 2026). Its incidence varies by CAR T-cell therapy construct, disease indication, pre-treatment conditioning, and patient-specific factors, with high-grade ICANS reported in up to 10% of cases in pooled analyses (Aziz et al., 2026). While not fully understood, its mechanism involves a complex interplay of systemic cytokine dysregulation, blood-brain barrier (BBB) disruption, and direct neuroinflammatory cascades within the central nervous system (CNS) (Aziz et al., 2026).

The current therapeutic backbone for ICANS management—corticosteroids—is effective in most although not all instances and can be associated with its own undesirable side effects (Aziz et al., 2026). As the field matures, there is an urgent need to develop new agents for steroid-refractory ICANS as well as steroid-sparing prophylactic and first-line therapeutic strategies with fewer adverse effects, grounded in mechanistic understanding of ICANS pathobiology (Aziz et al., 2026).

This perspective presents mitochondrial-targeted drugs including leflunomide, an immunomodulatory agent approved for rheumatoid arthritis and with an emerging mitochondrial pharmacology, as novel repurposing candidate for the management of ICANS. We describe the selection rationale, mechanistic hypotheses, existing data from analogous inflammatory CNS conditions, and a proposed roadmap for clinical evaluation.

## 2 Pathophysiology of ICANS: mechanistic overview

Understanding ICANS pathophysiology is essential for identifying rational therapeutic targets. ICANS is now conceptualized as a two-phase neurotoxicity process driven initially by systemic cytokine elevation and subsequently by CNS inflammatory amplification (Aziz et al., 2026).

### 2.1 Systemic immune dysregulation

Following infusion, robust CAR T-cell activation triggers the release of proinflammatory biomarkers and cytokines including lactate dehydrogenase (LDH), ferritin, c-reactive protein (CRP), interleukin-6 (IL-6), interferon-gamma (IFN-γ), IL-1β, granulocyte-macrophage colony-stimulating factor (GM-CSF), and tumor necrosis factor-alpha (TNF-α) (Frey and Porter, 2019; Xiao et al., 2021; Zandaki et al., 2025). Serum levels of these mediators correlate with ICANS severity and have been used to develop predictive biomarker models, such as the Endothelial Activation and Stress Index (EASIX) which incorporates LDH and its modified version (m-EASIX) which incorporates LDH and CRP (Zandaki et al., 2025; Su et al., 2024).

The cytokine surge from activated CAR T cells acts as the trigger for secondary activation of innate immune cells, particularly circulating monocytes and macrophages. One murine study demonstrated that CRS severity is mediated not by CAR T cell–derived cytokines, but by IL-6, IL-1, and nitric oxide produced by recipient macrophages (Giavridis et al., 2018). Norelli et al. showed in humanized mice that human monocytes were the major source of IL-1 and IL-6 during CRS, and that monocyte depletion abated the syndrome (Norelli et al., 2018). GM-CSF is a key CRS-promoting protein that drives monocyte differentiation and macrophage polarization toward a pro-inflammatory phenotype (Sachdeva et al., 2019). GM-CSF neutralization with lenzilumab reduces myeloid cell infiltration, neuroinflammation, and CRS in patient-derived xenograft models (Sterner et al., 2019).

### 2.2 Endothelial activation and blood-brain barrier disruption

Elevated systemic cytokines, particularly IL-6, IL-1β, and vascular endothelial growth factor, promote endothelial cell activation and increased vascular permeability (Kang et al., 2021). Endothelial activation is not merely a passive consequence of cytokine exposure but rather an active and self-amplifying process, characterized by upregulation of adhesion molecules such as intercellular adhesion molecule-1 (ICAM-1) and vascular cell adhesion molecule-1 (VCAM-1) (Hong et al., 2021). In 133 adults treated with CD19 CAR T cells, patients with severe neurotoxicity demonstrated evidence of endothelial activation and increased blood-brain-barrier (BBB) permeability, with the permeable BBB failing to protect the cerebrospinal fluid from high concentrations of systemic cytokines including IFN-γ (Gust et al., 2017). Circulating biomarkers of endothelial injury and activation, including angiopoietin-2, von Willebrand factor, and soluble thrombomodulin, have additionally been shown to correlate with ICANS severity, and their incorporation into clinical predictive indices such as EASIX further underscores the centrality of endothelial dysfunction in ICANS pathobiology (Gust et al., 2017; Galli et al., 2023; Korell et al., 2022). Gu et al. states that excessive IFN-γ exposure to pericytes causes them to produce IL-6 and vascular endothelial growth factor (VEGF), which exacerbates BBB dysfunction by disarraying the tight junctions between brain microvascular endothelial cells (Gu et al., 2022). This disruption of tight junction proteins allows systemic cytokines, activated immune cells, and potentially CAR T cells to penetrate the CNS compartment (Gu et al., 2022). This BBB breach is considered a critical early event in ICANS pathogenesis.

### 2.3 CNS neuroinflammation

Once systemic cytokines breach the BBB, they activate resident microglia and astrocytes within the CNS, perpetuating local cytokine production (Gu et al., 2022). Cerebrospinal fluid analysis from patients with ICANS reveals elevated IL-6, IL-8, IFN-γ, and interferon-gamma-induced protein 10 levels, confirming a compartmentalized neuroinflammatory milieu distinct from, though related to, the systemic cytokine storm (Gu et al., 2022). This local inflammatory activation leads to neuronal dysfunction, excitotoxicity, and in severe cases, cerebral edema (Gu et al., 2022).

The nature of microglial activation in this compartmentalized neuroinflammatory milieu is particularly consequential. Microglia, the resident macrophages of the CNS, shift from a homeostatic, ramified surveillant state to a reactively activated, pro-inflammatory phenotype, capable of autonomous local cytokine production independent of ongoing peripheral immune activity (Vinnakota et al., 2024). In B cell lymphoma mice, the TGFβ-activated kinase-1 (TAK1)-NF-κB-p38 mitogen-activated protein kinase (MAPK) pathway was activated in microglia after CAR19 T cell transfer (Vinnakota et al., 2024). This CNS myeloid amplification loop is a driver of both CRS and ICANS severity and forms the mechanistic basis for GM-CSF-directed interventions such as lenzilumab, currently under clinical investigation (Norelli et al., 2018; Sterner et al., 2019).

### 2.4 Mitochondrial involvement in neuroinflammation

An underappreciated dimension of ICANS pathobiology involves mitochondrial dysfunction. Neuroinflammatory cytokines—IL-1β, TNF-α, and IFN-γ—impair mitochondrial electron transport chain function, promote reactive oxygen species (ROS) generation, and trigger mitochondrial outer membrane permeabilization (MOMP), leading to apoptotic signaling in neurons and glial cells (Yang et al., 2007). Mitochondrial damage-associated molecular patterns (mtDAMPs), including mitochondrial DNA and formyl peptides, are potent activators of the nucleotide-binding domain, leucine-rich repeat family, pyrin domain containing 3 (NLRP3) inflammasome and toll-like receptor (TLR) 9 signaling, further amplifying neuroinflammation (Brooks et al., 2026). This bidirectional relationship between neuroinflammation and mitochondrial dysfunction creates a feedforward loop that may sustain and worsen ICANS independent of systemic cytokine levels, providing a mechanistically distinct and therapeutically targetable pathway.

In response to cytokine-driven mitochondrial stress, microglia can undergo metabolic reprogramming toward aerobic glycolysis, a shift associated with pro-inflammatory activation. Succinate, which accumulates as a consequence of broken tricarboxylic acid cycle (TCA) cycle activity, is oxidized at Complex II, driving reverse electron transport at Complex I and generating a burst of mitochondrial ROS (mtROS) (Nair et al., 2019; Peruzzotti-Jametti et al., 2024; Mills et al., 2016). This mtROS signal can stabilize hypoxia inducible factor (HIF)-1α, which in turn transcriptionally activates IL-1β and other pro-inflammatory mediators (Tannahill et al., 2013). Additionally, cytokine-induced mitochondrial membrane permeabilization in microglia releases mtDAMPs into the cytosol, activating the NLRP3 inflammasome and cyclic GMP-AMP synthase-stimulator of interferon genes (cGAS-STING) pathways (Xian et al., 2022). Furthermore, activated microglia can propagate mitochondrial damage-associated signals to neighboring neurons through tunneling nanotubes, exosomes, and paracrine cytokine secretion—extending the zone of mitochondrial neuroinflammatory injury beyond the initially activated cells (Henrich and Scheiblich, 2026).

Cytokine-mediated impairment of astrocytic mitochondrial function can reduce glutamate transporter (excitatory amino acid transporter 2/glutamate transporter 1 (EAAT2/GLT-1)) activity, leading to synaptic glutamate accumulation and excitotoxic neuronal injury (Sheng et al., 2013; Benarroch, 2024). Mitochondrial ROS in reactive astrocytes drives NF-κB activation and the production of complement components C3 and C1q, which have been shown to promote synapse elimination and neuronal death (Li et al., 2023; Lawrence et al., 2023). Additionally, astrocytic mitochondrial dysfunction compromises the metabolic support that astrocytes normally provide to neurons through the astrocyte-neuron lactate shuttle, exacerbating neuronal bioenergetic failure (Bonvento and Bolaños, 2021; Beard et al., 2022).

Cardiolipin is a dimeric phospholipid localized to the inner mitochondrial membrane, where it plays essential roles in stabilizing electron transport chain (ETC) complexes and regulating cytochrome c anchoring (Pizzuto and Pelegrin, 2020). Under conditions of mitochondrial oxidative stress, cardiolipin undergoes peroxidation by cytochrome c, is subsequently externalized to the outer mitochondrial membrane and ultimately released extracellularly (Li et al., 2025). Externalized oxidized cardiolipin serves as a potent NLRP3 inflammasome activator (Elliott et al., 2018) and has been shown to engage TLR4 on microglia, triggering NF-κB-mediated pro-inflammatory transcription (Pizzuto and Pelegrin, 2020). This positions cardiolipin peroxidation not merely as a marker of mitochondrial damage but as an active upstream driver of neuroinflammatory amplification.

## 3 Current management of ICANS

### 3.1 Grading and monitoring

ICANS is graded (1–5) using the ICANS grading scale, which incorporates the Immune Effector Cell-Associated Encephalopathy (ICE) score (0–10), level of consciousness, seizure activity, motor findings, and intracranial pressure (Lee et al., 2019). For neurotoxicity monitoring and early detection, patients are administered an ICE questionnaire on day 0 pre-CAR T-cell therapy administration and on clinical assessment up to day +30.

### 3.2 Prophylaxis: corticosteroids

Prophylactic corticosteroid strategies, typically dexamethasone administered at the time of CAR T-cell therapy infusion or early post-infusion, have been explored at various centers (Oluwole et al., 2021). Based on the results of Zuma-1 Cohort 6, for example, prophylactic dexamethasone administration on days 0, +1, and +2 of axicabtagene autoleucel was approved by the Food and Drug Administration (FDA) to reduce the risk of both CRS and ICANS (Oluwole et al., 2021). While some retrospective and prospective data suggest benefit in attenuating ICANS severity, concerns persist regarding the potential for steroids to impair CAR T-cell expansion and compromise therapeutic efficacy (Giles et al., 2018). Standardized prophylaxis protocols are not yet universally adopted, reflecting the field’s ongoing uncertainty about optimal risk-benefit balancing.

### 3.3 First-line treatment: corticosteroids

For established ICANS, corticosteroids are the mainstay treatment per the American Society for Transplantation and Cellular Therapy guidelines. While Grade 1–2 ICANS is typically treated with 10 mg dexamethasone doses, grade 3–4 ICANS requires escalation to 1000 mg methylprednisolone doses (Brudno and Kochenderfer, 2024; Le et al., 2018). Since ICANS may overlap with CRS, tocilizumab may need to be administered concomitantly. Serial neurological assessments, neuroimaging, neurological consultation, electroencephalography (EEG), and, in selected cases, lumbar puncture are integral to management (Brudno and Kochenderfer, 2024). Patients with high grade ICANS may require escalation of care to the intensive care unit for closer observation. The immunosuppression caused by glucocorticoids broadly dampens cytokine production and inflammatory gene transcription through various mechanisms, including Jak/STAT, glucocorticoid receptor, and TLR (Bianchi et al., 2000). While steroids are effective in most cases, a subset of patients develop steroid-refractory ICANS associated with poor outcomes (Frisch et al., 2026). Additional complications experienced by patients in clinical practice include steroid-induced hyperglycemia, acute steroid myopathy, and steroid-induced psychosis (Barker et al., 2023; Dubovsky et al., 2012). These medical problems can result in prolonged hospital stays and require longer recovery post therapy.

## 4 Alternative and investigational steroid-sparing approaches

Recognition of the limitations of steroid-based management has catalyzed the exploration of more targeted agents. Below are approaches under active investigation, organized by mechanistic class.

### 4.1 IL-6 pathway blockade: tocilizumab

Tocilizumab, a humanized anti-IL-6 receptor monoclonal antibody, has an expanded indication approval from the FDA for CRS management (Brudno and Kochenderfer, 2024; AVTOZMA, 2024; Associate, 2026). While effective for CRS, its efficacy in ICANS is less established. IL-6 receptor blockade may paradoxically increase CNS IL-6 levels due to impaired clearance, and clinical data have not demonstrated consistent benefit for ICANS specifically. Ongoing studies are clarifying its role and optimal timing (Hadley et al., 1987; Valtis and Park, 2024).

### 4.2 IL-1 blockade: anakinra

Anakinra, a recombinant IL-1 receptor antagonist, blocks both IL-1α and IL-1β signaling (Park et al., 2023). Preclinical models of CRS and ICANS implicate IL-1 as a central driver of myeloid activation and BBB disruption (Norelli et al., 2018; Gust et al., 2017). Retrospective and prospective pilot data suggest anakinra may reduce ICANS severity without compromising CAR T expansion (Park et al., 2023). Its small molecular size allows for CNS penetration, a potential pharmacokinetic advantage over larger biologics in the neuroinflammatory context (Park et al., 2023; Jai et al., 2023).

### 4.3 Intrathecal corticosteroids with or without intrathecal chemotherapy

For refractory to systemic steroids high-grade ICANS, intrathecal (IT) corticosteroid administration—typically IT dexamethasone or IT methylprednisolone—has been employed in case series and institutional protocols (Jai et al., 2023; Katsin et al., 2024). It is believed that direct CNS delivery of steroids may achieve higher local drug concentrations while minimizing systemic exposure, though robust prospective data remain limited. Steroids may be combined with chemotherapy—methotrexate and/or cytarabine. This approach has been evaluated in case series with promising efficacy by eliminating CAR T-cells and proinflammatory cytokines directly in the CNS. However, the impact of these therapies on CAR T-cell persistence in the CNS remains unknown (Meng et al., 2025).

### 4.4 Dasatinib

Dasatinib, a BCR-ABL/Src kinase inhibitor with established CNS penetrance, has emerged as an intriguing steroid-sparing candidate. Its ability to inhibit CAR T-cell signaling through Src kinase blockade provides a pharmacological ‘off-switch’ capable of attenuating both CRS and ICANS without systemic immunosuppression (Killock, 2019; Mestermann et al., 2019). Early clinical experience and ongoing trials suggest dasatinib may be effective in managing severe toxicity; however, at the cost of reducing CAR T cell activation and function (Weber et al., 2019).

### 4.5 Intravenous immunoglobulin

Intravenous Immunoglobulin (IVIG) has been employed empirically in ICANS management, particularly in cases with suspected autoimmune or antibody-mediated components. Its mechanisms include Fc receptor blockade, complement inhibition, and immune modulation via anti-idiotypic antibodies (Prasad et al., 1999; Schwab and Nimmerjahn, 2013; Nimmerjahn and Ravetch, 2008; Takai, 2002). Evidence is primarily anecdotal, but IVIG is generally well-tolerated and may offer benefit in specific ICANS subtypes (Mokhtari et al., 2025).

### 4.6 TNF-alpha antagonists

Given the central role of TNF-α in neuroinflammation, TNF-α antagonists including infliximab and etanercept have been proposed as steroid-sparing agents for refractory ICANS (Canadian agency for drugs and technologies in health, 2026). TNF-α directly activates microglia, promotes BBB permeabilization, and contributes to excitotoxic neuronal injury (Olmos and Lladó, 2014; Montgomery and Bowers, 2012; Li et al., 2022). Infliximab, is a large chimeric IgG1 monoclonal antibody that has poor BBB permeability, however, making it not a suitable candidate for directly targeting the CNS (Dadsetan et al., 2016).

### 4.7 Other cytokine-directed agents

Additional cytokine-targeting approaches under investigation include siltuximab (anti-IL-6), canakinumab (anti-IL-1β), emapalumab (anti-IFN-γ), and ruxolitinib (JAK1/2 inhibitor targeting downstream cytokine signaling) (Li et al., 2022; Mulvey et al., 2025). Each addresses distinct nodes in the ICANS cytokine network, and combination or sequential strategies may ultimately be required for refractory cases.

### 4.8 Next-generation CAR T constructs

Beyond pharmacological intervention, next-generation CAR T-cell engineering strategies aim to reduce inherent toxicity potential. These include: (1) incorporation of inducible safety switches enabling controlled CAR T elimination (2) use of alternative signaling domains (e.g., CD28 versus 4-1BB co-stimulation); with differential cytokine secretion profiles; (3) armored CAR T cells co-expressing anti-inflammatory payloads; (4) logic-gated or Boolean CARs designed for tumor selectivity; and (5) fully humanized single-chain variable fragment domains that minimize immunogenicity (Killock, 2019; Mestermann et al., 2019; Krause et al., 1998; Wang et al., 2025). CRISPR-engineered products with cytokine gene knockouts (e.g., GM-CSF disruption) have demonstrated reduced toxicity in preclinical and early clinical settings (Yi et al., 2021).

### 4.9 Lymphodepleting chemotherapy regimens

Traditional lymphodepleting regimens including fludarabine carry risk of neurotoxicity. Clinical investigators explored alternative chemotherapies such as bendamustine during an international fludarabine shortage but those were found to be not as effective (Bharadwaj et al., 2024; Chahine et al., 2024). Additionally, scientists are actively exploring chemotherapy-free approaches to CAR T cells as well as *in-vivo* CARs which do not require lymphodepletion (Volta et al., 2026).

## 5 Mitochondrial-targeted drug repurposing for ICANS: rationale, selection framework, and a prototype example

### 5.1 Mitochondria at the intersection of inflammation and neurotoxicity

Mitochondria are increasingly recognized as central regulators of innate immune signaling and inflammatory amplification—far beyond their classical role as ATP-generating organelles (Weinberg and Chandel, 2025). In the context of neuroinflammation, mitochondrial dysfunction operates as both a downstream consequence of cytokine-driven injury and an upstream driver of sustained inflammatory cascades, creating a feedforward loop that is particularly relevant to ICANS pathobiology (Weinberg and Chandel, 2025).

Under homeostatic conditions, mitochondria tightly regulate ROS production, calcium buffering, and apoptotic signaling. During acute neuroinflammation, cytokines such as IL-1β, TNF-α, and IFN-γ impair ETC complex I and III activity, leading to excessive mtROS generation (Qin et al., 2024). Oxidative stress causes lipid peroxidation, protein carbonylation, and mitochondrial DNA (mtDNA) damage, ultimately triggering mitochondrial outer membrane permeabilization (MOMP) and the release of damage-associated molecular patterns (DAMPs) (Weinberg and Chandel, 2025).

Mitochondrial DAMPs—including mtDNA, formyl peptides, cytochrome C, and ATP—are potent activators of innate immune receptors. mtDNA activates cGAS-STING and TLR9 signaling, driving type I interferon responses and NF-κB-mediated transcription of proinflammatory cytokines (Kim et al., 2023; Tao et al., 2024). Extracellular ATP signals through P2X7 purinergic receptors, a major priming and activation signal for the NLRP3 inflammasome (Amores-Iniesta et al., 2017). NLRP3 activation drives caspase-1-mediated processing and secretion of IL-1β and IL-18, cytokines centrally implicated in ICANS severity (Martínez-Reyes and Chandel, 2020). Critically, this entire cascade can be perpetuated autonomously within the CNS compartment by activated microglia and astrocytes, independent of ongoing systemic cytokine elevation—potentially explaining why ICANS may persist or worsen after peripheral cytokine control is achieved (Weinberg and Chandel, 2025).

Beyond innate immune signaling, mitochondrial dysfunction shapes the metabolic phenotype of infiltrating and resident immune cells in ways that determine their inflammatory output. Activated M1-polarized macrophages and microglia rely on aerobic glycolysis (the Warburg effect) and exhibit a broken tricarboxylic acid cycle with accumulation of succinate and itaconate. Succinate oxidation by mitochondrial complex II drives reverse electron transport and mtROS generation, amplifying IL-1β production (Martínez-Reyes and Chandel, 2020). Interventions that restore or redirect mitochondrial metabolism toward oxidative phosphorylation (OXPHOS) have been shown to attenuate M1 polarization and promote anti-inflammatory phenotype transitions. These metabolic checkpoints represent tractable pharmacological targets distinct from, and complementary to, conventional cytokine blockade (Algieri et al., 2025).

Neurons are uniquely vulnerable to mitochondrial dysfunction given their high bioenergetic demand and limited glycolytic capacity (Yusuf et al., 2025). Neuronal mitochondria serve critical roles in synaptic vesicle trafficking, calcium homeostasis, and axonal transport (Yusuf et al., 2025). Cytokine-mediated mitochondrial impairment in neurons may disrupt these functions, contributing to excitotoxicity, synaptic dysfunction, and ultimately the clinical neurological manifestations of ICANS. Targeting the mitochondrial axis, therefore, offers the potential to protect neurons directly—a neuroprotective dimension absent from current steroid-based or cytokine-blocking strategies (Algieri et al., 2025; Yusuf et al., 2025).

### 5.2 Framework for selecting mitochondrial-targeted repurposing candidates

The drug repurposing paradigm—leveraging approved or clinically advanced compounds with established safety and pharmacokinetic profiles for new indications—offers a pragmatic and accelerated pathway to address the unmet need for steroid-sparing ICANS management. Repurposed mitochondrial-targeting drugs are particularly appealing because their mechanisms act upstream of, or in parallel with, the cytokine cascades targeted by existing investigational agents, offering genuinely differentiated and potentially synergistic activity.

**FIGURE 1:**
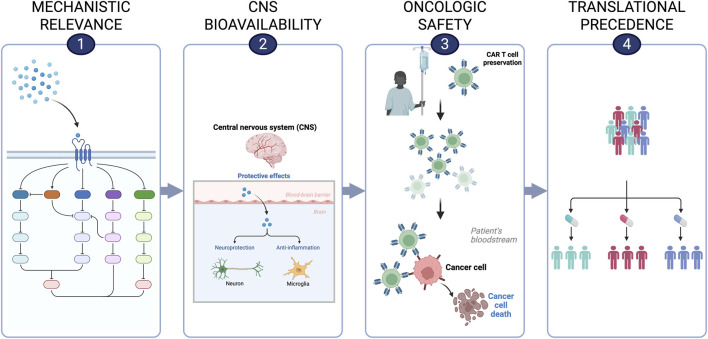
Selection Criteria for Mitochondrial-Targeted Drug Repurposing Candidates for ICANS Management in CAR T-cell Therapy. 1. Mechanistic relevance: The drug must directly modulate one or more mitochondrial targets implicated in neuroinflammation—including dihydroorotate dehydrogenase (DHODH), Complex I/III of the ETC, mtROS scavenging, NLRP3 inflammasome activation, cGAS-STING signaling, or mitochondrial biogenesis pathways (e.g., PGC-1α, AMPK). 2. CNS bioavailability: Adequate BBB penetrance is essential. Candidates were prioritized if CNS exposure had been demonstrated pharmacokinetically, either through prior clinical measurement (cerebrospinal fluid levels) or shown efficacy in clinical neurodegenerative and/or inflammatory models. 3. Oncologic Safety: A critical differentiator from corticosteroids. Candidates with mechanisms likely to impair CAR T-cell expansion or effector function were penalized unless a pharmacological rescue for the drug or dosing strategy could mitigate this concern. 4. Translational precedent: Evidence of activity in disease models with mechanistic overlap to ICANS—including neuroinflammatory diseases (rheumatoid arthritis, multiple sclerosis, autoimmune encephalitis), immune effector cell toxicities (graft-versus-host-disease, hemophagocytic lymphohistiocytosis), or cytokine-driven inflammatory syndromes—was weighted favorably. Created in BioRender. Zahedi, A (2026) https://BioRender.com/2sscrjk.

We applied a structured, multi-criteria selection framework to identify mitochondrial-targeting repurposing candidates for ICANS. Candidate drugs were required to satisfy criteria across four domains: mechanistic relevance, CNS bioavailability, oncologic safety, and translational precedent (Figure 1).

Application of this framework generated a candidate landscape that includes but is not limited to:

- inhibitors of DHODH (ex: leflunomide/teriflunomide);
- mitochondrial-targeted antioxidants (ex: SS-31/elamipretide);
- NLRP3 inflammasome inhibitors with mitochondrial activity (ex: MCC950, OLT1177);
- AMPK activators that promote mitochondrial biogenesis and anti-inflammatory metabolic reprogramming (ex: metformin);
- and cGAS-STING pathway inhibitors with mitochondrial upstream effects.

Each candidate occupies a distinct mechanistic niche within the mitochondrial neuroinflammatory axis and warrants systematic preclinical and early clinical evaluation. None, to our knowledge, are currently under formal investigation for ICANS prophylaxis or treatment, representing a genuinely open therapeutic space.

### 5.3 Prototype example: leflunomide

To illustrate how the above framework translates to a specific candidate, we highlight leflunomide as a prototype repurposing example—one that satisfies all four selection criteria and has a particularly well-developed translational rationale for ICANS treatment.

Leflunomide is an orally bioavailable isoxazole derivative, FDA-approved for rheumatoid arthritis and psoriatic arthritis. Its active metabolite, teriflunomide (A77 1726), inhibits DHODH, a mitochondrial inner membrane enzyme essential for *de novo* pyrimidine synthesis (National Center for Biotechnology Information, 2026). By depleting intracellular uridine, teriflunomide selectively suppresses rapidly proliferating lymphocytes incapable of compensating via salvage pathways. This DHODH-centered mechanism satisfies criterion 1 through its direct mitochondrial enzymatic target and downstream effects on mtROS, NLRP3 inflammasome priming, and macrophage metabolic polarization.

Regarding CNS bioavailability (criterion 2), teriflunomide is FDA approved for relapsing multiple sclerosis (MS), providing direct clinical evidence of CNS penetrance and neuroinflammatory efficacy (National Center for Biotechnology Information, 2026). Cerebrospinal fluid teriflunomide levels have been measured in MS clinical trial populations, confirming pharmacologically relevant exposure in the CNS compartment—an advantage that many larger biologics under ICANS investigation cannot claim.

For oncological safety (criterion 3), leflunomide’s long-term safety record in patients with rheumatoid arthritis is well-characterized. Leflunomide’s most common potential side effects are hepatotoxicity, which requires monitoring and is manageable with dose adjustment, and teratogenicity, which necessitates standard contraceptive counseling. Importantly, leflunomide has demonstrated antiviral activity against cytomegalovirus—a clinically meaningful added benefit in the post-CAR T immunosuppressed setting (Gokarn et al., 2019).

With respect to CAR T-cell activity preservation, the primary concern is that DHODH inhibition could impair CAR T-cell expansion. However, this risk is mitigable: DHODH inhibition is reversible, preferentially affects highly proliferating cells, and can be pharmacologically rescued by pyrimidine (uridine) supplementation if needed. Careful timing of leflunomide initiation relative to CAR T infusion—and potentially brief dosing holidays during the critical expansion window—represent straightforward design elements in a clinical protocol.

Translational precedent (criterion 4) is based on variety of conditions. Beyond MS, leflunomide has been explored in neuropsychiatric SLE (featuring BBB disruption and cerebrospinal fluid cytokine elevation), graft-versus-host disease (an immune effector cell toxicity with mechanistic parallels to ICANS), and viral encephalitis (National Center for Biotechnology Information, 2026; Vijaysekharan et al., 2018; Ong et al., 2014).

Collectively, this body of evidence establishes leflunomide as a mechanistically coherent, clinically tractable prototype for the broader class of mitochondrial-targeted ICANS repurposing candidates.

**FIGURE 2:**
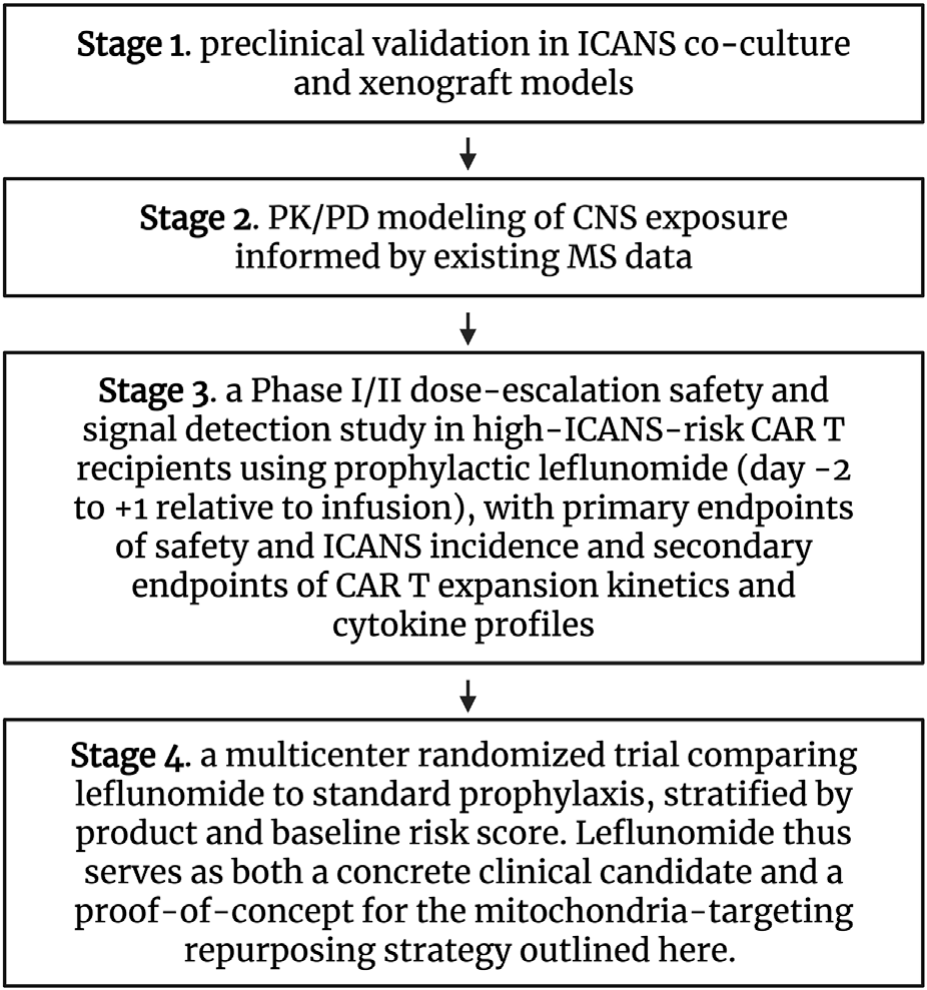
A multi-stage approach to validating leflunomide as a prophylaxis drug for ICANS in CAR T-cell therapy. Created in BioRender. Zahedi, A (2026) https://BioRender.com/cm9jaig.

A proposed evaluation pathway for leflunomide for ICANS prophylaxis in CAR T-cell therapy is presented in Figure 2. It lists the recommended steps for assessment of the drug, from pre-clinical studies to a randomized clinical trial comparing leflunomide to standard-of-care approaches.

## 6 Future directions

The broader ICANS management landscape is rapidly evolving, and several future directions merit attention.

Biomarker-driven prophylaxis represents a priority area: validated predictive models incorporating pre-infusion ferritin, CRP, and LDH, as well as CAR T-product-specific expansion kinetics may allow risk stratification to guide prophylactic intervention intensity, including steroid-sparing regimens like leflunomide. Development of CNS-specific inflammatory biomarkers—whether serum-based (e.g., GFAP, NfL, S100B) or cerebrospinal fluid-derived—will facilitate earlier diagnosis and treatment response assessment.

Combination steroid-sparing strategies targeting non-redundant pathways—for instance, DHODH inhibition combined with IL-1 blockade or TNF-α antagonism—may offer additive or synergistic benefit compared to single-agent approaches. Adaptive clinical trial designs that incorporate biomarker-stratified randomization and response-adaptive dosing will be essential to efficiently evaluate such combinations.

Artificial intelligence-driven drug repurposing pipelines may offer a complementary and scalable approach to candidate identification beyond leflunomide. Network pharmacology analyses mapping drug-target-disease relationships across large databases (DrugBank, ChEMBL, OpenTargets) serve to identify candidate agents for CRS and ICANS that await experimental validation. Integrating transcriptomic signatures from ICANS patient biopsies with drug perturbation databases (CMap, LINCS) represents a hypothesis-generating strategy to systematically expand the steroid-sparing candidate pipeline.

Finally, the development of universally applicable, low-toxicity CAR T platforms—including allogeneic off-the-shelf products engineered with intrinsic toxicity attenuation features—may ultimately reduce ICANS incidence at the source, complementing pharmacological management strategies. The convergence of improved cell engineering, biomarker-guided risk stratification, and mechanistically targeted steroid-sparing pharmacotherapy offers a realistic path toward a future in which ICANS is reliably prevented or managed without the immunosuppressive complications of high-dose corticosteroids.

## 7 Conclusion

ICANS remains a clinically significant barrier to the full realization of CAR T-cell therapy’s curative potential and safe outpatient administration. Current steroid-based management is empiric, broadly immunosuppressive, and inadequate in a meaningful proportion of patients. A growing portfolio of steroid-sparing agents under investigation—including tocilizumab, anakinra, dasatinib, IT steroids with or without IT chemotherapy, IVIG, and TNF-α antagonists—reflects the field’s recognition that more targeted, mechanism-based approaches are needed.

We propose that mitochondrial-targeting drugs represent an underexplored but mechanistically compelling class of repurposing candidates for ICANS management. The mitochondrial axis—encompassing ETC., dysfunction, mtROS generation, mtDAMP-driven innate immune activation, NLRP3 inflammasome engagement, and metabolic polarization of microglia—constitutes a feedforward neuroinflammatory loop that current steroid-based and cytokine-blocking strategies do not adequately address. Targeting this axis offers neuroprotective potential absent from existing approaches, and the drug repurposing framework allows this hypothesis to be tested with established agents whose safety and pharmacokinetics are already well-characterized.

Among the candidate landscape identified through our multi-criteria selection framework—including DHODH inhibitors, mitochondrial-targeted antioxidants, NLRP3 inhibitors, AMPK activators, and cGAS-STING inhibitors—leflunomide emerges as a prototype with uniquely favorable translational credentials: direct mitochondrial enzymatic targeting via DHODH, proven CNS anti-inflammatory activity, manageable safety in immunocompromised populations, and a pharmacological rescue strategy for CAR T-cell preservation. A staged evaluation pathway from preclinical validation to randomized clinical trial is feasible and warranted. This perspective aims to catalyze both the experimental investigation of leflunomide and the broader program of mitochondrial-targeted repurposing for ICANS management.

## Data Availability

The original contributions presented in the study are included in the article/supplementary material, further inquiries can be directed to the corresponding author.
